# Data Dashboard Acceptability, Use, and Perceived Effectiveness in Disseminating Local Overdose Data and Resources in a Rural New York State County: A Cross-Sectional Study

**DOI:** 10.2196/68977

**Published:** 2025-07-10

**Authors:** Zhongxuan He, Monika Salvage, Corinna A Noel

**Affiliations:** 1 Department of Public and Ecosystem Health, Cornell University, 350 Tower Road, Ithaca, NY, 14853, United States; 2 Department of Mental Health, Cayuga County, Auburn, NY, United States

**Keywords:** public health data dashboard, opioid-related disorders prevention and control, data-driven decision making, data visualization, rural health, community resources

## Abstract

**Background:**

In 2023, Cayuga County, a rural county in New York State, developed and published a publicly available, interactive overdose dashboard highlighting demographic, geographic, and time trends in suspected overdoses as well as substance use–related resources in the community. Despite the widespread use of data dashboards in the overdose crisis, there is little evidence to suggest that these dashboards can effectively disseminate data and enable public health data-driven decision-making, especially in a rural county. We conducted an evaluation of the Cayuga County Overdose Data Dashboard to fill this knowledge gap.

**Objective:**

Our study aimed to evaluate the Cayuga County Overdose Data Dashboard’s acceptability, use, and perceived effectiveness in disseminating overdose data and resources.

**Methods:**

Following the launch of the dashboard, an online Qualtrics survey collected feedback from individuals older than 18 years of age living or working in Cayuga County, asking respondents to reflect upon their experience using the dashboard. The 10-minute survey assessed usage patterns and motivations to access the dashboard as well as the dashboard’s ease of use, most valued design features, and overall perceived effectiveness in communicating information on overdoses and local resources. Data were analyzed using descriptive statistics.

**Results:**

From May to December 2023, a total of 61 individuals from Cayuga County completed the survey, including those with lived substance use experience (n=8, 13%) as well as their close contacts (n=28, 46%), health care providers (n=12, 20%), law enforcement (n=11, 18%), and local public health and mental health care professionals (n=27, 44%). The user-friendly design and frequent updates facilitate engagement, as 54% (n=33) of respondents reported accessing the dashboard at least monthly and 75% (n=46) using it to inform decision-making. Most thought that the dashboard was easy to use (n=59, 97%) and very effective in disseminating information (n=46, 76%). From the 8 different types of overdose-related information portrayed on the dashboard, the most valued were the locations of treatment and recovery services, scoring an average of 4.75 (SD 0.65) on a 5-point scale (1=”Not important” to 5=”Most important”), followed by the locations of free, publicly accessible Naloxone (mean 4.58, SD 0.89) and trends in fatal and nonfatal overdoses (mean 4.48, SD 0.81).

**Conclusions:**

Overall, this study suggests that the Cayuga County Overdose Data Dashboard effectively disseminates information and enables data-driven decision-making in the region. When developing a community-level dashboard, our findings underscore the necessity of a user-friendly design, frequent data updates, and inclusion of key information and visuals on local overdose trends and resources.

## Introduction

The overdose crisis is a continuously evolving public health concern, remaining one of the leading causes of death among adults in the United States [1-3]. From 2002 to 2022, the age-adjusted rate of fatal overdoses quadrupled from 8.2 to 32.6 per 100,000 population, driven by increases in overdoses involving opioids and, more recently, fentanyl [1,4,5]. Following a 30% spike observed during the COVID-19 pandemic, fatal overdose rates have plateaued, although deaths among certain population subgroups, like adults older than 35 years, have increased [3]. Overdoses in New York State (NYS) mirror the national trends [3]. In 2021, NYS reported 69.4 opioid-related emergency department (ED) visits and 25.3 opioid-related deaths per 100,000 residents [6]. Our study region of Cayuga County, a heavily impacted rural area in NYS, ranked in the top quartile statewide in 2021 in ED usage with 85.6 opioid-related ED visits and 26.4 opioid-related deaths per 100,000 residents [6].

As the overdose crisis continues to evolve, a localized, data-driven approach has been suggested to be effective, using real-time data to track overdose-related trends and identify high-need populations and regions to target public health interventions [7-10]. Many data dashboards have been developed with the goal of disseminating actionable data on overdoses [9,11,12]. Previous research indicates that for a dashboard to be ’actionable,' it should address a specific information need, be easy to navigate, and offer local granularity [13]. However, only a minority of public health dashboards meet these criteria [14]. Consequently, the utility of existing overdose data available to practitioners is often hindered by missing, delayed, and nongranular data, failing to provide detailed insights on high-risk populations, regions, or substances [8,10]. For example, the NYS Department of Health publishes aggregate, county-specific, and opioid-related data on a quarterly basis [15], but these data are lagged up to 6 months and contain no information on demographics, substances beyond opioids, or locations where overdoses are occurring within the county. Furthermore, while features like hotspot maps have been shown to facilitate more targeted and effective intervention strategies [9], they are often absent from data dashboards.

An internal assessment of NYS county-level dashboards revealed significant gaps in local data availability, granularity, and functionality. As of April 2024, only 15 out of 62 (24.2%) NYS counties had a publicly accessible, county-specific overdose data dashboard or similar tool. Among these, 60% included demographic information (most frequently age and gender), 40% provided geographic details on overdose locations (by zip code or hotspot maps), and only 26.7% reported suspected substances involved in overdoses. Although few (11.3%) of these dashboards are updated monthly or more frequently, they offer a timelier alternative to state-level data. These findings underscore the need for more comprehensive, specific, and frequently updated overdose data dashboards to support regional public health interventions.

Since the COVID-19 pandemic, there has been a high demand for the rapid development of public health data dashboards worldwide [13,16,17]. These tools are essential for disseminating critical public health information, yet evaluations of overdose-specific dashboards remain limited. Our work contributes to the literature in this field. While previous studies often focus purely on technical and operational aspects of public health dashboards, specific design features such as data visualizations or ease of use [16,18], there is an evidence gap regarding the ability of data dashboards to effectively disseminate information and support decision-making in the overdose crisis, especially in a rural setting [18].

To address the need for timely and granular local overdose data to inform public health action, Cayuga County developed an overdose data dashboard in early 2023. Following its launch, we conducted a comprehensive evaluation of the dashboard, collecting feedback from a diverse sample of community members, including those who work in the substance use and mental health fields. As such, our evaluation aimed to answer the following questions: (1) How is the publicly available, community-level overdose data dashboard used? (2) Is the overdose data dashboard easy to use and understand? (3) Which design features and data indicators are valued on a community-level overdose data dashboard? (4) How effective is the overdose data dashboard in disseminating overdose-related data and resources?

## Methods

### Overview

In collaboration with Cayuga County Mental Health and a diverse group of community partners, we developed and launched a county-specific overdose data dashboard in early 2023 using the Shiny web application framework for R (RStudio, PBC) and evaluated its acceptability, use, and perceived effectiveness in disseminating local overdose data and resources using an online survey. Data collection ran from May to December 2023.

#### Development of the Data Dashboard

#### Data Used in Dashboard

The data used in this dashboard is obtained from ODMAP (Overdose Detection Mapping Application Program). ODMAP is a web-based platform developed by the High-Intensity Drug Trafficking Area to facilitate near real-time overdose surveillance and response at the county level [19]. In Cayuga County, the Auburn Police Department, the Cayuga County Sheriff’s Office, and the New York State Police provide information on each suspected overdose to which they respond. This includes the day and time, location, age, gender, and suspected substance, as well as whether the incident was fatal or nonfatal and if Naloxone was administered.

#### Dashboard Design

The need for a tool to disseminate granular and timely overdose–related data arose from discussions during monthly committee meetings focusing on substance use in Cayuga County. The committee, including approximately 20 volunteer representatives from county mental and public health, peer organizations, health care providers, law enforcement, community services, the court system, and higher education, collaborated with an epidemiologist (CN) from 2022 to 2023 to develop a county-specific overdose dashboard using ODMAP data. Intended end users of the dashboard included committee members, their peers, and members of the public.

An iterative design process was taken to prioritize the ease of use, clarity, and utility of the dashboard, with the goal of enabling data-driven decision-making. The initial selection of dashboard indicators and visuals was based on previously developed static monthly reports using ODMAP data. Committee members provided verbal and written feedback at least 4 times throughout the development process. The epidemiologist presented the most up-to-date draft dashboard in meetings and made changes based on this feedback. Comments often focused on ensuring the anonymity of those experiencing overdoses, suggesting improvements to support intended uses, and simplifying and clarifying visuals.

**Figure 1.**
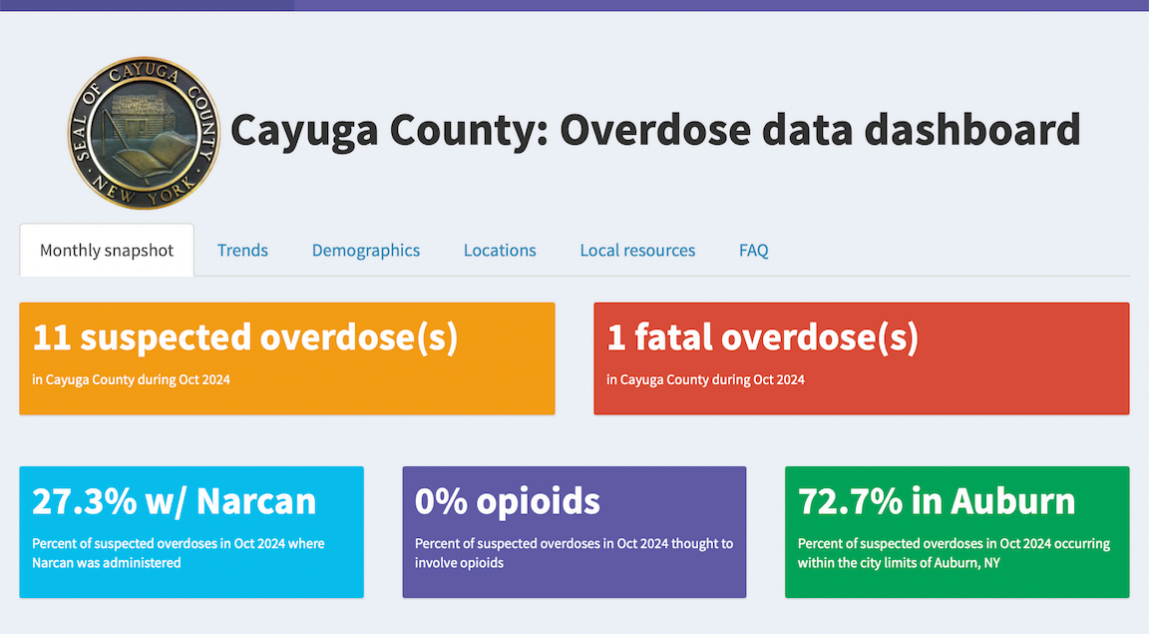
The “monthly snapshot” tab of the Cayuga County overdose data dashboard.

The final version of the dashboard was launched in March 2023 and is available on the county website. Developed using RShiny, it includes aggregate data on key overdose indicators, including a summary of the previous month, trends over the past several years (fatal and nonfatal overdoses, incidents where Naloxone was administered, and suspected substances), key demographics, and geographic locations of suspected overdoses as well as interactive features, such as customized visualizations, data filtering, and a choropleth map visualizing overdose hotspots (Figure 1 and Multimedia Appendix 1). The dashboard also provides information and maps portraying local resources, including publicly available naloxone boxes, local treatment and recovery services, and drug disposal drop boxes within the county.

### Evaluation of the Data Dashboard

Following the launch of the dashboard, we conducted a cross-sectional study targeting adults older than 18 years who lived or worked in Cayuga County using a 20-question online Qualtrics survey [20].

### Participant Recruitment

We conducted 2 phases of participant recruitment, resulting in a convenience sample. During the initial recruitment phase, spanning from May to August 2023, participants were offered a US $10 Amazon gift card as an incentive. They were recruited through direct links to the survey from the dashboard, a local digital newspaper segment highlighting the dashboard’s launch, and a social media advertisement on county mental health accounts that included a QR code and link to the dashboard and survey. We halted the first phase of recruitment due to concerns about fraudulent responses. To filter out fraudulent responses, respondents were required to complete a supplementary verification survey to confirm the demographic information provided in the initial survey. We excluded the respondents who provided differing information on key demographics for the original and subsequent verification survey. This initial recruitment yielded 21 valid responses. The second phase of recruitment targeted community members who worked in substance use or mental health fields through email invitations to community partners and flyers with QR codes posted in public locations (county buildings and offices of substance use–related organizations) as well as an increased incentive (US $20). This approach yielded 40 more valid responses between September and December 2023. In both phases, we excluded survey responses from those who indicated that they lived outside of Cayuga County and neighboring counties.

### Online Survey

The survey tool (see Multimedia Appendix 2) was designed to capture participant usage and motivations as well as perceptions of the dashboard’s ease of use and effectiveness in disseminating information. First, respondents provided informed consent and were prompted to navigate to the dashboard before beginning the survey. Usage and motivations were assessed by asking respondents how, how often, and for what reasons they accessed the dashboard, how they used the information, and how frequently they thought the dashboard should be updated. Perceived effectiveness of dissemination of data and resources was evaluated using 2 items on a 5-point Likert scale (5=“Very effective” to 1=“Not effective”), asking respondents to rate how effective the dashboard was in: (1) “sharing data on overdoses in the county” and (2) “sharing information on resources related to substance use in the county.” Respondents also indicated whether they thought they could find and access resources using the dashboard (5=“Strongly agree” to 1=“Strongly disagree”). Perceived value of the types of information portrayed was assessed using a 5-point scale (5=“Very important” to 1=“Not important”). User experiences related to navigation and features, such as interactivity, visuals, usefulness, ability to locate the dashboard, and loading time were also evaluated on a 5-point scale (5=“Strongly agree” to 1=“Strongly disagree”). We used the 2-item Usability Metric for User Experience Lite (UMUX-Lite) scale as an alternative to the widely used System Usability Scale (SUS) to assess the usability and acceptability of the dashboard [21]. The UMUX-Lite has been shown to be reliable (coefficient α of 0.86), sensitive, and valid, strongly correlating with the SUS (*r*=0.83) and the Net Promoter Score (NPS; *r*=0.72) and predicting SUS scores with 99% accuracy [22,23]. The UMUX-Lite score is calculated from 2 items on a 7-point scale (7=“Strongly agree” to 1=“Strongly disagree”): (1) “The functionalities of the data dashboard fulfill my needs” and (2) “The data dashboard is easy to use” [22-24]. A single-item Net Promoter Score was also included to assess user satisfaction and likelihood of recommending the dashboard to others [24]. In the last part of the survey, respondents provided information on their age, gender, race/ethnicity, education, home residence, role in the community, and background related to substance use.

We implemented several security measures to maintain data integrity via the Qualtrics platform: (1) the “Prevent Ballot Box Stuffing” option to prevent multiple entries from the same individual and (2) the built-in “reCAPTCHA” and “Bot Detection” algorithms to filter out automated bot responses [20,25]. Only responses with a reCAPTCHA score of >0.5 were included.

### Statistical Analysis

Descriptive statistics, including counts and percentages, and mean, SD, and range were used to summarize the study population as well as answer the primary evaluation questions. We used the regression formula proposed by Lewis et al [22] to calculate the predicted SUS from the UMUX-Lite survey items and subsequently categorized these scores based on letter grades (A-F) [22,23,26]. In exploratory analyses, we used Fisher exact tests to examine the association between the reported frequency of use of the dashboard and roles in the community, motivation for visiting the dashboard, and uses of dashboard information. Welch 2-tailed t test was used to assess the association among continuous ratings for usability, perceived value of data indicators, and user experiences related to navigation and design features, and categorical variables evaluating roles in the community, information use, and motivations. Data was analyzed in Stata (StataCorp), and a threshold of *P*<.05 was used to determine statistical significance [27].

### Ethical Considerations

The evaluation protocol was reviewed by the Cornell University institutional review board (protocol number IRB0147362) and was deemed to be “not research” as specified by the Department of Health and Human Services Code of Federal Regulations 45 CFR 46. Informed consent was obtained for all participants.

## Results

### Study Population

A total of 61 people provided feedback on the dashboard through the online survey, representing a well-educated, primarily female, and White population between the age groups of 35 and 64 (mean 42, SD 4.5, range 19-70) years, with diverse roles in the community (Table 1). All participants either lived or worked in Cayuga County. Most identified as family or friends of an individual with substance use (n=28, 46%) or as working in the substance use field (n=27, 44%).

**Table 1.** Study population.

Demographic Characteristics	Values, n (%)Total (N=61)	
Age (years)		
18‐24	4 (7)	
25‐34	13 (21)	
35‐44	23 (38)	
45‐64	18 (30)	
65‐84	3 (5)	
Gender		
Woman	43 (71)	
Man	18 (30)	
Race		
White or Caucasian	61 (100)	
Education		
Some high school, but no degree	3 (45)	
High school diploma or GED^a^	4 (67)	
Associate degree or some college	21 (34)	
Bachelor’s degree	20 (33)	
Postgraduate degree	13 (21)	
Role in community^b^		
A person with lived experience of substance use	8 (13)	
A family member/friend of someone with substance use experience	28 (46)	
A health care provider	12 (20)	
An individual who works with organizations that address mental health and/or substance use	27 (44)	
A member of law enforcement	11 (18)	
A first responder	5 (8)	
A community member	26 (43)	

a GED: General Educational Development.

b Total may add up to >100% because individuals could indicate more than one role.

### Dashboard Use

Most respondents (n=42, 69%) had accessed the overdose dashboard prior to participating in our online survey, with 54% (n=33) visiting the dashboard monthly or more frequently, for a variety of reasons, on both mobile devices and computers (Table 2). Notably, 75% (n=46) of respondents used the dashboard to inform organizational or personal decision-making, and 44% (n=27) advocated for change related to overdose prevention and treatment. Respondents cited wanting to stay informed about overdose trends (n=44, 72%), applying it in their work (n=26, 43%), and using it to monitor the impact of overdose-related interventions (n=15, 25%). Only 8% (n=5) of respondents reported using it to find resources related to substance use.

**Table 2.** Reported dashboard usage.

Variable	Values, n (%)Total (N=61)	
Dashboard visit frequency		
Daily	3 (45)	
Weekly	9 (15)	
Monthly	21 (34)	
Yearly	9 (15)	
Never	19 (31)	
Device typically used to visit the dashboard		
Mobile device	16 (26)	
Computer/laptop	20 (33)	
Both mobile device or computer/laptop	11 (18)	
Motivations for visiting dashboard^a^		
To stay informed about overdose trends in Cayuga County	44 (72)	
To inform my work as a health care provider, first responder, or other community stakeholder	26 (43)	
To monitor the impact of interventions aimed at reducing overdoses	15 (25)	
To find resources related to substance use for myself or someone I know	5 (8)	
Reported use of information on dashboard^a^		
To inform my own actions or decision-making	34 (53)	
To advocate for change related to overdose prevention and treatment	28 (44)	
To inform the actions or decision-making of the organization for which I work	24 (39)	
To support grant writing or fundraising efforts for which organization I work	8 (13)	
I don’t use the information on the dashboard for any reason	7 (12)	

a Categories may add up to >100% because individuals could indicate more than one option.

Exploratory analyses revealed that reported visit frequency varied based on self-identified roles in the community as well as motivations for visiting the dashboard (Table 3). Community members and close contacts of individuals with substance use experience accessed the dashboard more frequently compared to other respondents (community members *P*=.007, close contacts: *P*=.03), as did those who reported wanting to stay informed about overdose trends (*P*=.02). Those who wished to monitor the impact of overdose-related interventions also tended to access the dashboard more frequently, although this was not statistically significant (*P*=.09). This contrasts with members of law enforcement, who accessed the dashboard less frequently (*P*<.05). Dashboard visit frequency did not vary substantially based on how participants reported using the information (*P*>.05).

**Table 3.** Factors influencing dashboard usage.

Factor	Dashboard usage (%)				*P* value	Values, n (%)
	Weekly or more often	Monthly	Yearly	Never		
Role in community						
a person with lived experience of substance use	50	12.5	12.5	25.0	.13	8 (13)
a family member or friend of someone with lived experience of substance use	35.7	39.3	7.1	17.9	.007	28 (46)
a health care provider	16.7	50.0	25.0	8.3	.15	12 (20)
an individual who works with organization(s) that address mental health and/or substance use	17.2	37.9	17.2	27.6	.86	29 (48)
a member of law enforcement	9.1	9.1	18.2	63.6	.05	11 (18)
a first responder	20.0	20.0	20.0	40.0	.86	5 (8)
a community member	34.6	38.5	7.7	19.2	.03	26 (43)
Motivation^a^						
To stay informed about overdose trends in Cayuga County	22.7	43.2	13.6	20.5	.02	44 (72)
To monitor the impact of interventions aimed at reducing overdoses	33.3	46.7	0.0	20.0	.09	15 (25)
To inform my work as a health care provider, first responder, or other community stakeholder	23.1	38.5	15.4	23.1	.71	26 (42.6)
To find resources related to substance use for myself	33.3	33.3	33.3	0.0	1	3 (4.92)
To find resources related to substance use for someone I know, such as a friend, family member, or colleague	50.0	50.0	0.0	0.0	.78	2 (3.28)
Use of information^a^						
To inform my own actions or decision-making	29.2	33.3	16.7	20.8	.36	24 (38.3)
To inform the actions or decision-making of the organization that I work for	18.8	46.9	12.5	21.9	.16	32 (52.5)
To support grant writing or fundraising efforts for the organization I work for	37.5	25.0	12.5	25.0	.68	8 (13.1)
To advocate for change related to overdose prevention and treatment	22.2	44.4	11.1	22.2	.37	27 (44.3)
I do not use the information presented on the dashboard for any reason	—^b^	—	—	—	—	7 (11.5)

a Categories may add up t o>100% because individuals could indicate more than one option.

b Not applicable.

### User Experience

The mean predicted SUS score was 79.5 (SD 8.4; range: 39.2-87.9), indicating that the Cayuga County Overdose Data Dashboard neared the eightieth percentile of SUS responses and was within the “satisfactory” range [28]. A total of 88% (n=54) of respondents rated the dashboard above a grade “B” based on the curved grading scale interpretation of the SUS score, suggesting the dashboard is perceived as a “good” product [28]. Looking at the individual items of the UMUX-Lite scale, respondents rated the dashboard as easy to use (mean 6.2, SD 0.8; range 3-7) and able to meet their needs (mean 6.3, SD 0.8; range 2-7). Those who used the dashboard to inform organizational decision-making, on average, rated the usability of the dashboard higher compared to those not using the dashboard for this purpose (*P*<.001), with a mean UMUX-Lite rating of 89.84 (SD 8.12) compared to 83.91 (SD 16.20), respectively.

The dashboard received an overall positive NPS of 21.3, with 43% (n=26) of respondents classified as promoters, 36% (n=22) as passive, and 21% (n=13) as detractors. This aligns with the predicted SUS score, suggesting that users are satisfied with the dashboard’s capabilities. Despite 31% (n=19) of respondents reporting having never accessed the dashboard before participating in our survey, all (n=61, 100%) now report intending to visit the dashboard in the future.

### Valued Features in Dashboard Design

Overall, the information portrayed on the dashboard and selected design features were highly rated by respondents (Table 4). Of the 8 different types of overdose-related information and resources portrayed on the dashboard, the most valued were the locations of treatment and recovery services, followed by the locations of free, publicly accessible Naloxone, and aggregate trends in fatal and nonfatal overdoses. All items were rated above a 4 on a 5-point Likert scale on average, indicating a high level of perceived importance for all. Survey respondents thought the data portrayed were useful (mean 4.3, SD 0.69; range 3-5), and were confident that they would be able to find and access naloxone (mean 4.3, SD 0.72; range 2-5), treatment and recovery services (mean 4.2, SD 0.78; range: 1-5), and medication drop boxes (mean 4.2, SD 0.78; range 2-5) in the community if needed. In open-ended feedback, some users suggested that the list of recovery resources could be expanded to include other resources beyond naloxone, medication drop-boxes, and peer, treatment, and recovery services.

Exploratory analyses suggest that close contacts of individuals with substance use experience tend to perceive suspected substances involved in overdoses as more important to portray on a dashboard compared to other respondents, with mean ratings of 4.64 (SD 0.67) and 4.30 (SD 0.68), although this was not statistically significant (*P*=.06). Mean ratings on dashboard design did not appreciably differ by reported role in the community (*P*>.05).

**Table 4.** Data indicators and design features incorporated on dashboard.

Data indicators and design features	Mean (SD)Total (N=61)
Perceived importance of information portrayed on the dashboard^a^	
Locations of treatment and recovery services	4.8 (0.65)
Locations of free naloxone	4.6 (0.89)
Trends in fatal and nonfatal overdoses	4.5 (0.81)
Trends in suspected substances involved in overdoses	4.5 (0.70)
Locations of medication drop boxes	4.4 (0.89)
Trends in naloxone administration at scenes of overdoses	4.4 (0.74)
Overdose statistics by age and gender	4.4 (0.83)
Overdose statistics of the previous month	4.4 (0.84)
Overdose statistics by geographic area	4.3 (0.80)
Dashboard design^b^	
The types of data portrayed on the dashboard are useful.	4.3 (0.69)
The interactive nature of the dashboard makes it easy for me to find the information that I am looking for.	4.3 (0.59)
Based on the information provided on the dashboard, I can easily find and access naloxone if needed.	4.3 (0.73)
The visuals have enough descriptive text for me to understand what they are showing.	4.2 (0.58)
Based on the information provided on the dashboard, I can easily find and access medication drop boxes if needed.	4.2 (0.78)
Based on the information provided on the dashboard, I can easily find and access treatment and recovery services if needed.	4.2 (0.79)
The dashboard is easy to find on the Cayuga County website.	4.1 (0.71)
The dashboard webpage takes too long to load.	2.6 (1.11)

a Data portrays mean rating and SD of “How important do you think it it for the dashboard to show each type of information?” on 5-point Likert scale (5=Very important to 1=Not important).

b Data portrays mean rating and SD of “Based on your experience using the dashboard, how much do you agree or disagree with each of the following statements?” on 5-point Likert scale (5=Strongly agree to 1=Strongly disagree).

### Ability to Disseminate Information

The majority of respondents (n=46, 75%) reported that the dashboard was effective in disseminating information on both overdose statistics (mean 4.13, SD 0.90) and substance use resources (mean 4.05, SD 0.96) in the county. Ratings did not considerably differ by reported roles in the community (*P*>.05).

## Discussion

### Principal Findings

Our results suggest that the Cayuga County Overdose Data Dashboard is a valuable, effective, and useful tool for disseminating information on overdose trends and resources available in this rural community. Compared to the publicly available NYS Department of Health data, our dashboard provides more timely updates and insights into overdose trends and resources available regionally. Among the 42 respondents who used the dashboard prior to our study, 78.6% (n=33) of respondents reported accessing it monthly or more frequently, indicating consistent use in this group. Users from a wide variety of backgrounds generally report that the dashboard is easy to use and understand, with the information on the dashboard used for a variety of purposes including to inform personal or organizational decision-making, support grant writing, and/or advocate for change related to overdose prevention and treatment. In our sample, 19 out of 61 respondents had never seen the dashboard prior to completing the evaluation survey, which highlights the need for broader outreach to increase awareness and use in the community.

As overdose data dashboards become more prevalent, understanding how end users perceive and use the information is important for enhancing their public health impact. This study evaluates specific indicators, design features, and use of an overdose data dashboard, offering insights into how tools can be adapted to meet needs in similar rural settings. This work also highlights the potential of county-level data dashboards to drive action in underserved areas.

### Insights and Best Practices for Developing Effective Overdose Dashboards

#### Selection of Indicators and Visualization of Data

Our evaluation provides valuable insights for entities wishing to develop overdose dashboards, particularly in terms of the types of information presented and how the integrated data are visualized. The Cayuga County Overdose Data Dashboard includes information on key demographics (age and gender) as well as geographic locations of overdoses and local substance use–related resources, in addition to monthly, year-to-date, and annual counts in nonfatal and fatal overdoses, naloxone administration, and suspected substances involved in overdoses. The elements included on the dashboard were identified during the development process with frequent feedback from a diverse group of community partners and are corroborated by research, which emphasizes the importance of tracking and visualizing overdose data by location, time, and population while preserving anonymity for small communities [29,30]. The Council of State and Territorial Epidemiologists has suggested best practices for visualizing jurisdictional overdose data, which include choosing the right type of chart (eg, line charts for trends and bar charts for comparisons), keeping visuals simple and clear, using a minimal color palette, and labeling all elements clearly to enhance understanding and decision-making [31]. Based on previous recommendations to highlight overdose hotspots and resources using the geographic information system [9], overdose hotspots on our dashboard are depicted with a choropleth map based on population-normalized overdose rates within town boundaries, while resources are disseminated through a map pinpointing selected resources in the community, accompanied by a list with their corresponding addresses and contact information. While some research advocates for a theory-driven approach to dashboard design [18], we opted for a less formal, community-driven approach, emerging naturally from discussions during monthly substance-use committee meetings.

#### Inclusion of Location-Specific Information on Resources

Our findings indicate that dashboard users value and find all the information portrayed on the dashboard useful, but especially location-specific information on substance use–related resources, which they thought they could find and access if needed, based on the information provided on the dashboard. Some users suggested that the list of recovery resources on the dashboard could be expanded to include additional services beyond naloxone, medication drop-boxes, and peer, treatment, and recovery services. This aligns with findings from other studies, which highlight that incorporating local information such as links to substance-use resources and interactive maps with features down to the neighborhood level enhances user engagement and promotes action [30,31]. Our results suggest that it is important to not only share summary statistics and trends on publicly available public health data dashboards but also actionable information on resources that community members may be able to benefit from. To be “actionable,” the resources portrayed should be relevant to users, detailed enough to allow users to find and access resources locally, and the information on resources should be easy to find and navigate to on the dashboard [13,14].

#### Planning for Diverse User Needs

We highlight that public health dashboards, particularly related to the overdose crisis, should plan for and accommodate diverse user needs. In our survey, 45.9% (n=28) of respondents reported multiple roles in the community, from community members to individuals working within substance use–related organizations to close contacts of people with lived experience. This highlights the need for a multifaceted and flexible dashboard interface, portraying multiple types of data to be able to meet these diverse needs. Respondents cited both personal and work-related reasons for accessing the dashboard, including a desire to be aware of the overdose trends and to inform action and decision-making around substance use. Despite these varying motivations, respondents indicated that the dashboard was able to fulfill their needs, potentially a result of early involvement of end users in the design process, as has been previously suggested [32]. Respondents also indicated that the interactive nature of the dashboard enables them to find the information they are looking for. In the Cayuga County Overdose Data Dashboard, interactivity is facilitated using dropdown menus, allowing users to filter visuals and summary statistics by date range and indicator of interest, as well as maps showing geographic locations of overdoses and resources, where users can hover their mouse to get location-specific information. This is in line with prior work, which indicates that effective data dashboards facilitate data exploration and are adaptable to a wide range of needs, workflows, and devices, stressing the importance of customizable filters that allow users to focus on the information that is most relevant to them [9,33].

#### Emphasis on User-Friendly Design

Our work underscores the importance of user-friendly design in promoting engagement with data dashboards. We use both the UMUX-Lite and NPS, which are well-established tools in the health care and technology fields and provide efficient and reliable measures of usability, user satisfaction, and loyalty [23,24]. To our knowledge, this is one of the first applications to use both tools to evaluate public health overdose data dashboards, reducing participant burden while providing actionable and comparable insights, making them ideal instruments for our purposes. The favorable UMUX-Lite and NPS ratings by respondents suggest that our dashboard is user-friendly and likely to be recommended to others [24]. With a predicted SUS score of 79.5, the Cayuga County Overdose Data Dashboard is perceived as more favorable when compared to other recently developed overdose data dashboards [34]. Notably, those who used the dashboard to inform organizational decision-making rated the usability of the dashboard higher than other respondents. We attribute the perception of the dashboard as user-friendly to the early and frequent feedback received from a diverse group of community partners throughout the development process [32]. As noted above, prior work suggests the use of a theory-driven approach to enhance usability, which future dashboards could consider as a guiding principle in their design process [18].

### Limitations

There are several limitations to our study. With a relatively small convenience sample of 61 participants, our findings may not represent the views of all those who live or work in Cayuga County. However, we were able to recruit a diverse sample of dashboard users from varied backgrounds. Results are only generalizable to overdose dashboards serving primarily rural regions and may not be applicable to dashboards developed for other public health problems. Given our recruitment strategy, we were unable to calculate a response rate, which introduces the risk of and our ability to assess selection bias. In addition, while we present several exploratory analyses, our study was not powered to examine differences in use, acceptability, and ability to disseminate information to individuals with different roles in the community or reasons for accessing the dashboard. Future research should examine the interplay between our outcomes and differing sociodemographics, including occupations and motivations for dashboard use. Our study focused on Cayuga County, a rural area with a predominantly White population. Due to the absence of race and ethnicity data in ODMAP, these important social constructs were not included in the dashboard or assessed in our evaluation. Future overdose data dashboards should incorporate detailed demographic data whenever possible, including race and ethnicity, to better identify disparities and guide targeted public health interventions [35,36]. In addition, while our findings show that respondents value all data indicators and rate navigation features highly, it remains unclear which specific elements in the dashboard promote action and how users leverage the information to make decisions.

### Recommendations for Cayuga County

While the findings highlight the value of the Cayuga County Overdose Data Dashboard, they also reveal areas for improvement. First, outreach efforts to both the public and those working locally in the substance use and mental health fields should be increased to raise awareness and usage of the dashboard. Second, expanding the list of recovery, substance use, and social service resources could provide more comprehensive support to those that need it. Finally, incorporating more detailed demographic data may help identify disparities and guide interventions for vulnerable populations.

### Conclusions

Customizable and user-friendly data dashboards such as the Cayuga County Overdose Data Dashboard represent a promising tool to effectively disseminate timely data and enable regional decision-making in the overdose crisis. These tools can support personal efforts (such as finding treatment resources, securing naloxone, or advocating for change) as well as organizational decision-making (like implementing targeted interventions), and assist in securing additional overdose-related grant funding for the region. When developing a community-level dashboard, our findings underscore the necessity of early engagement of end users as well as inclusion of aggregate statistics to describe overall trends, interactive visuals that highlight high-need populations and regions, and maps with actionable information on resources in the community.

## Supplementary material

10.2196/68977 Multimedia Appendix 1 Screenshots of the Cayuga County Overdose Data Dashboard.

10.2196/68977 Multimedia Appendix 2 Survey instrument.

## Abbreviations

ED: emergency department
NPS: Net Promoter Scale
NYS: New York State
ODMAP: Overdose Detection Mapping Application Program
SUS: System Usability Scale
UMUX-Lite: Usability Metric for User Experience-Lite
