# Supplementary material for: Data Dashboard Acceptability, Use, and Perceived Effectiveness in Disseminating Local Overdose Data and Resources in a Rural New York State County: A Cross-Sectional Study
Source: Online J Public Health Inform. 2025 Jul 10;17:e68977. doi: 10.2196/68977 (PMC12270186; doi:10.2196/68977)

Cayuga County Overdose Data Dashboard
Available: https://www.cayugacounty.us/1634/Overdose-Data-Dashboard

Supplemental Figure 1: Screenshot of the "Monthly snapshot" tab

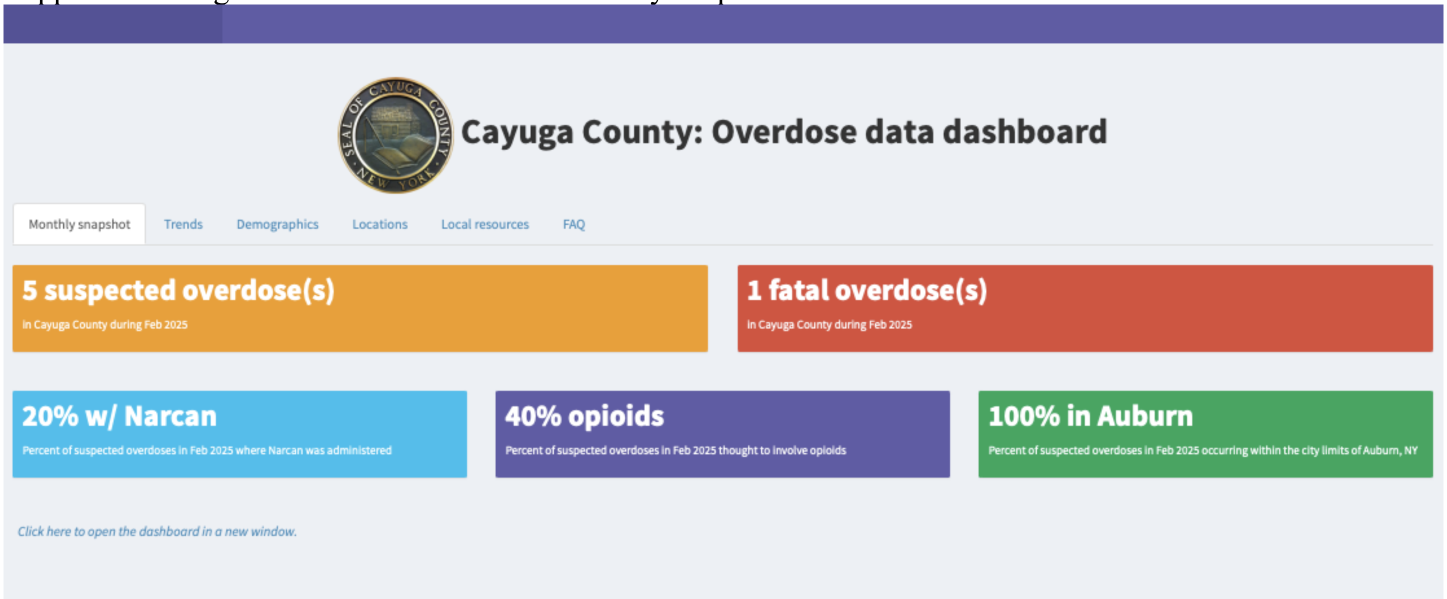

Supplemental Figure 2: Screenshot of the "Trends" tab

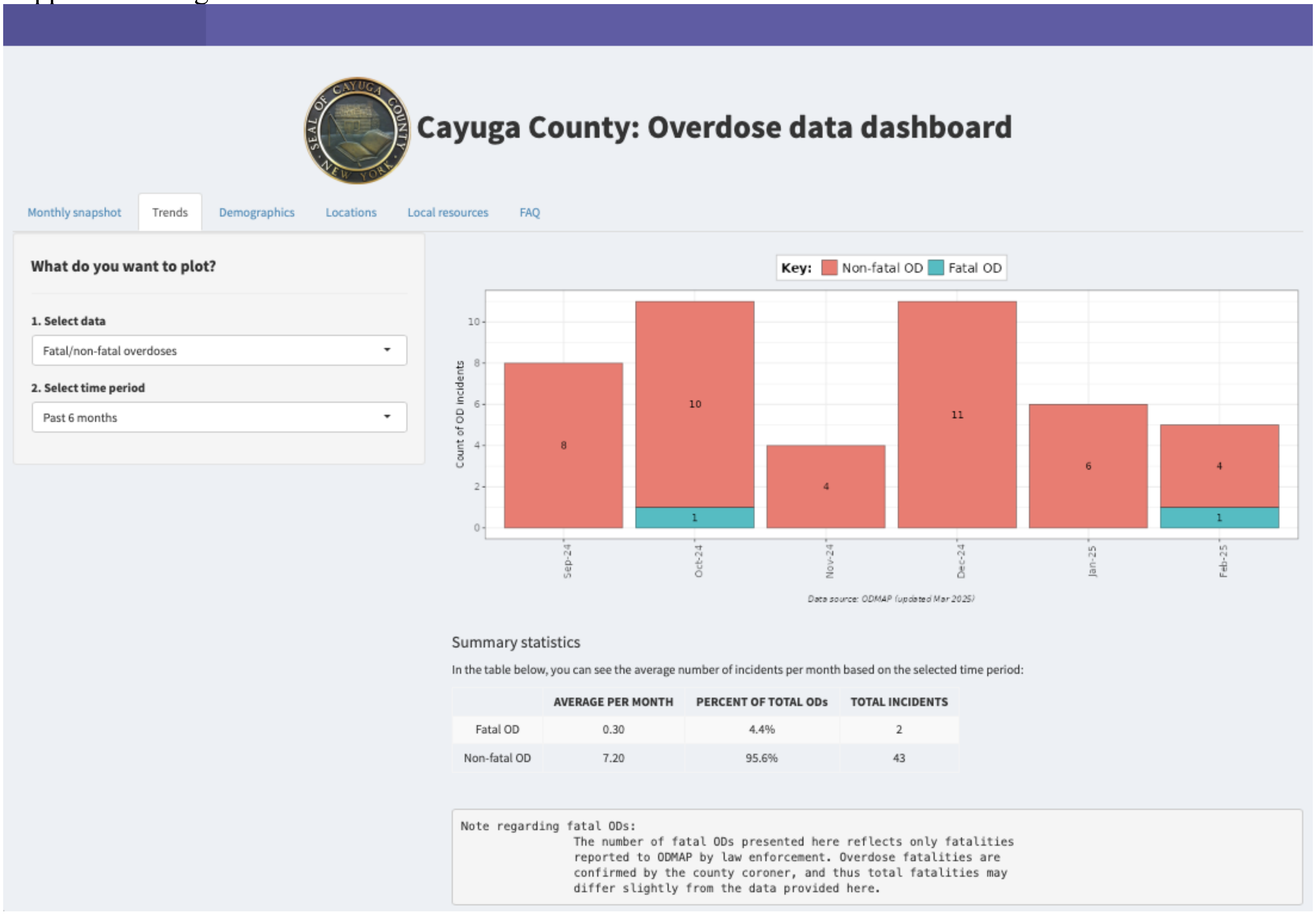

Supplemental Figure 3: Screenshot of the “Demographics” tab

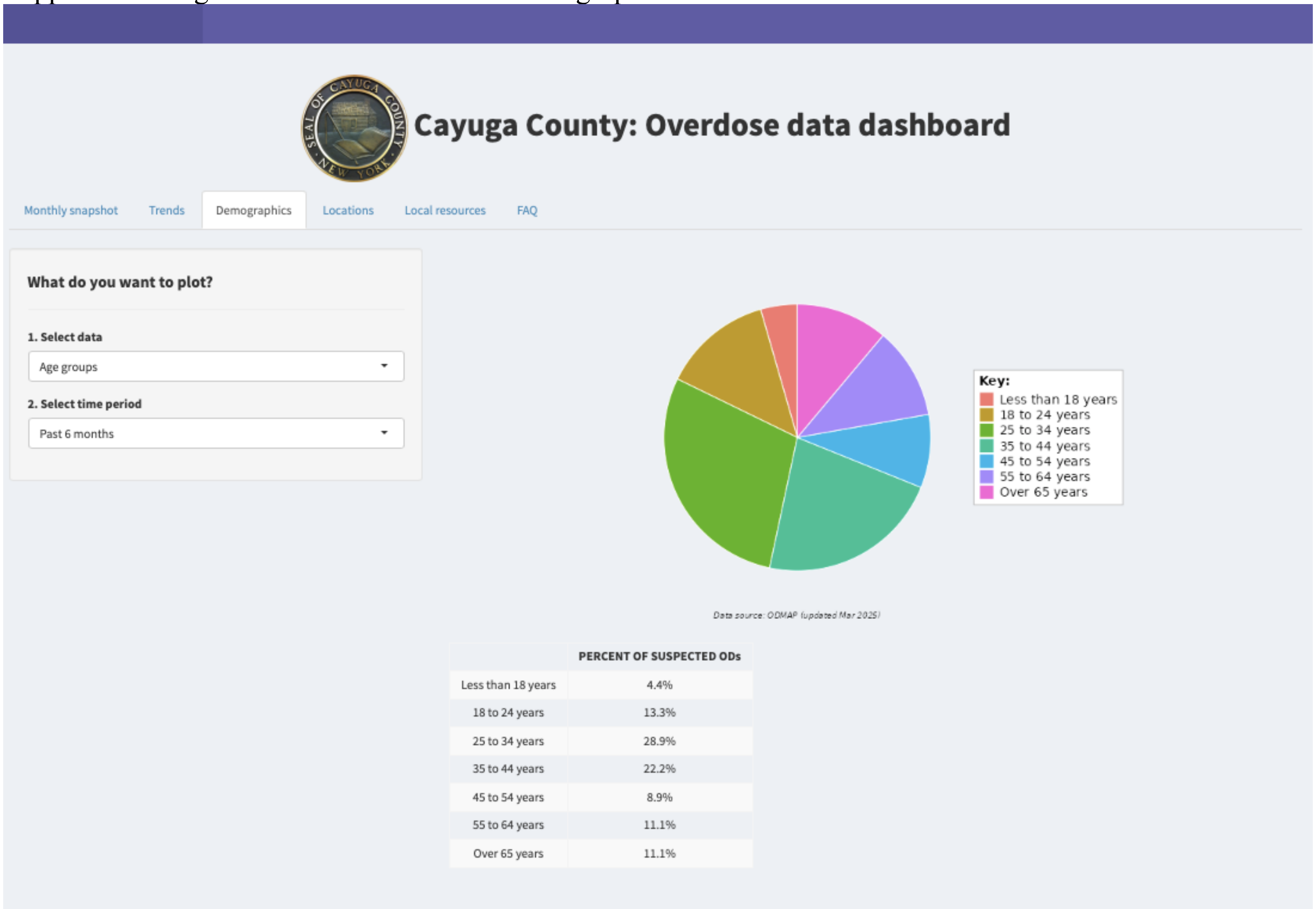

Supplemental Figure 4: Screenshot of the “Locations” tab

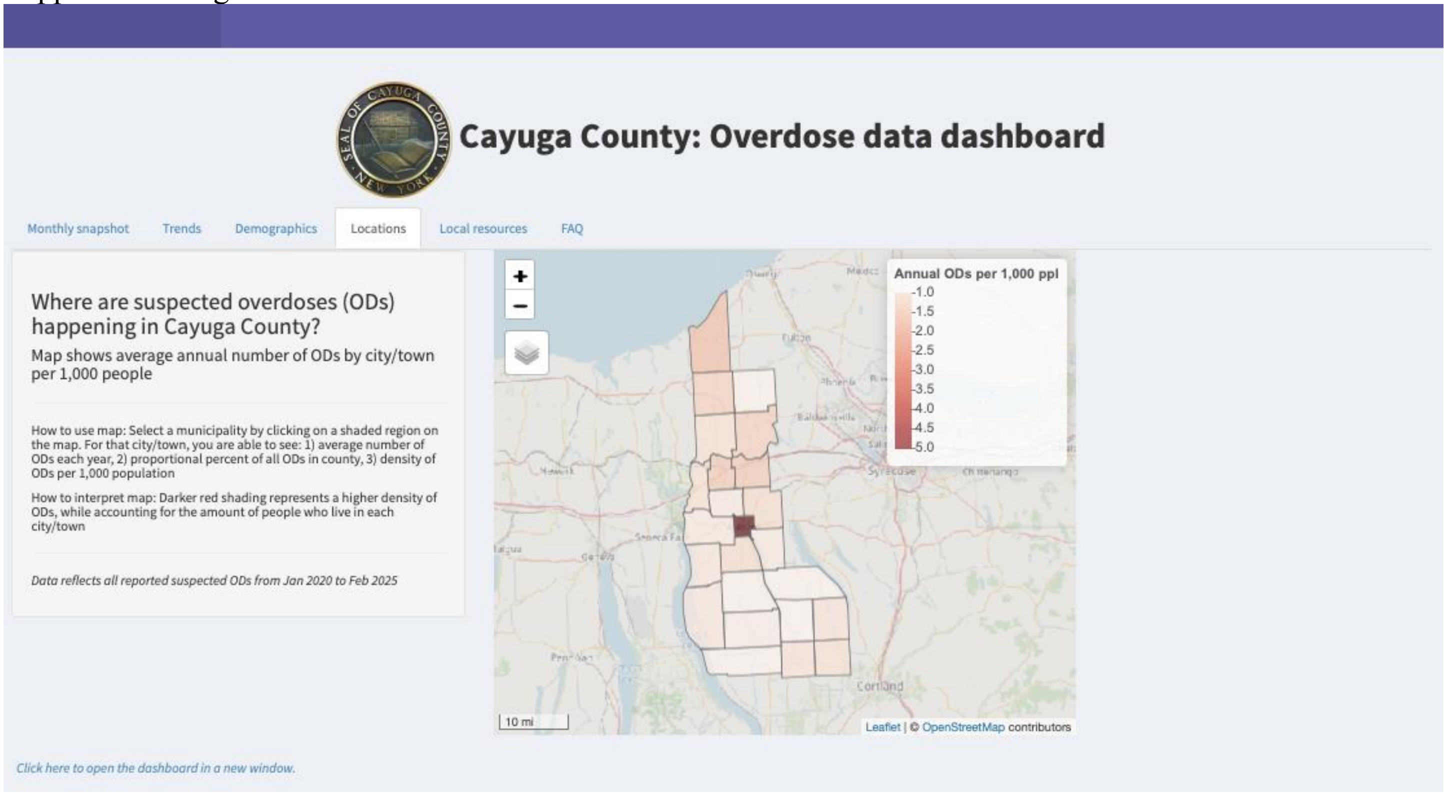

Supplemental Figure 5: Screenshot of the “Local resources” tab

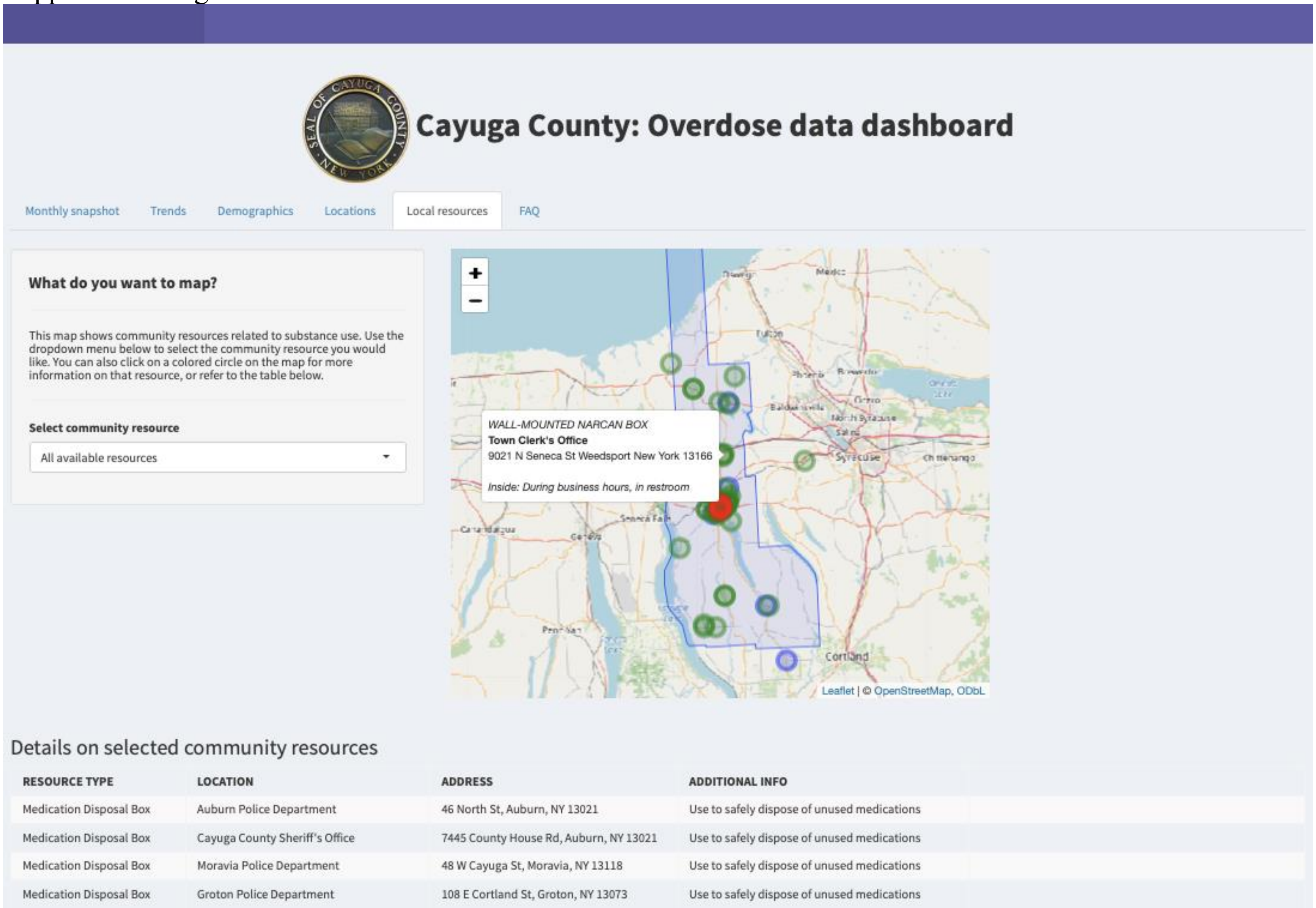

Supplemental Figure 6: Screenshot of the “FAQ” tab

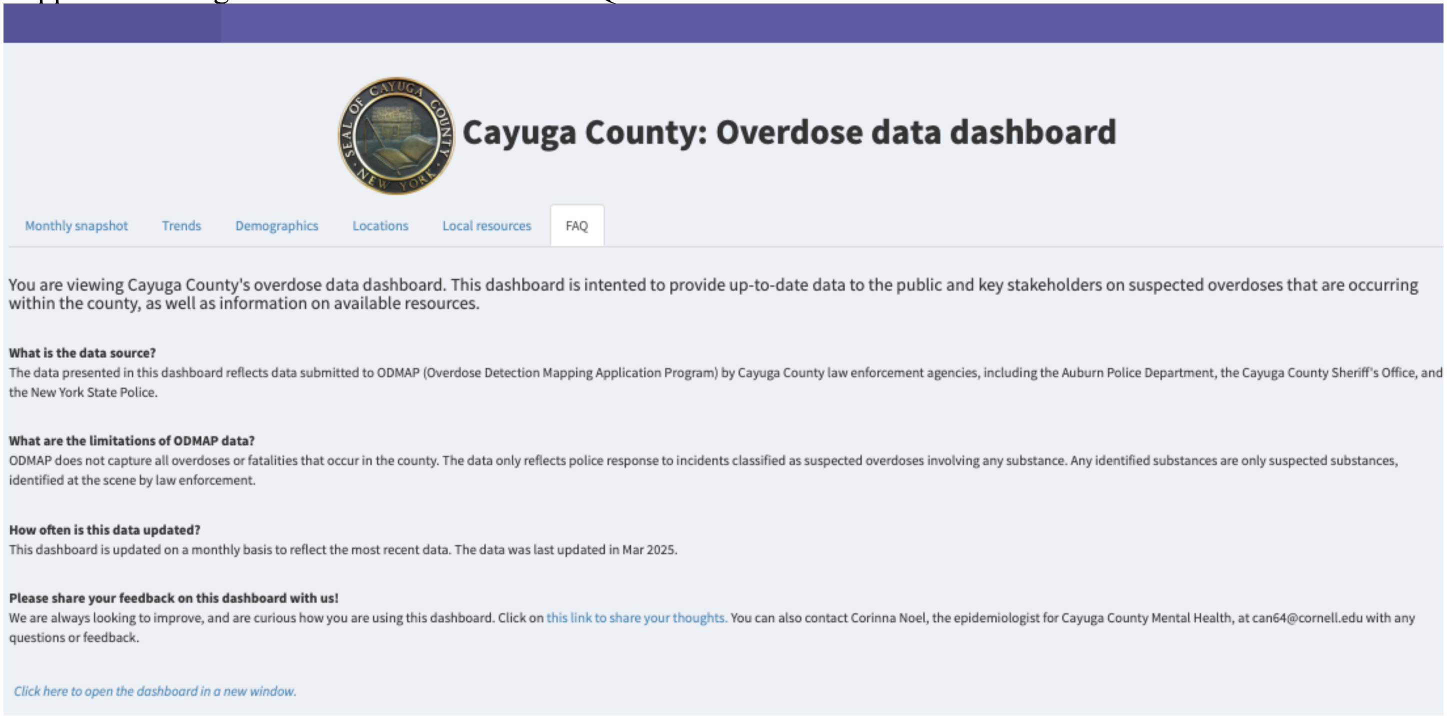

Supplement: Multimedia Appendix 1 [file ojphi-v17-e68977-s001.pdf]
