# Supplementary material for: Data Dashboard Acceptability, Use, and Perceived Effectiveness in Disseminating Local Overdose Data and Resources in a Rural New York State County: A Cross-Sectional Study
Source: Online J Public Health Inform. 2025 Jul 10;17:e68977. doi: 10.2196/68977 (PMC12270186; doi:10.2196/68977)
Supplement: Multimedia Appendix 2 [file ojphi-v17-e68977-s002.pdf]

Part 1

We will first ask you questions about how you use the Cayuga County Overdose Data Dashboard. If you are not already familiar with the dashboard, please take some time to familiarize yourself prior to beginning the survey via this link: https://www.cayugacounty.us/1634/Overdose-Data-Reports-Trends

1. How often do you access the Cayuga County Overdose Data Dashboard?

   Daily
   Weekly
   Monthly
   Yearly
   Never

2. What are your reason(s) for visiting the Cayuga County Overdose Data Dashboard? *(check all that apply)*

   To stay informed about overdose trends in Cayuga County
   To monitor the impact of interventions aimed at reducing overdoses
   To inform my work as a healthcare provider, first responder, or other community stakeholder
   To find resources related to substance use for myself
   To find resources related to substance use for someone I know, such as a friend, family member, or colleague
   Other, please specify ____

3. How do you use the information from the Cayuga County Overdose Data Dashboard? *(check all that apply)*

   To inform my own actions or decision-making
   To inform the actions or decision-making of the organization that I work for
   To advocate for change related to overdose prevention and treatment
   I do not use the information presented on the dashboard for any reason
   Other (please specify) _____________

4. Based on your needs, how often should the information on the Cayuga County Overdose Data Dashboard be updated?

   Daily
   Weekly
   Monthly
   Yearly

5. Overall, how effective do you think the Cayuga County Overdose Data Dashboard is in...?

	Very effective	Effective	Fairly effective	Slightly effective	Not effective
Sharing data on overdoses in the county	○	○	○	○	○
Sharing information on resources related to substance-use in the county	○	○	○	○	○

Part 2

We would like to understand which types of information you value on the Cayuga County Dashboard. If you wish to review the dashboard prior to answering the below questions, you can find the link to the dashboard here: https://www.cayugacounty.us/1634/Overdose-Data-Reports-Trends.

6. The following types of information are currently presented on the Cayuga County Overdose Data Dashboard. How important do you think it is it for the dashboard to show each type of information?

	Very important	Important	Moderately important	Slightly important	Not important
Overdose statistics of the previous month	○	○	○	○	○
Trends in fatal and non-fatal overdoses	○	○	○	○	○
Trends in suspected substances involved in overdoses	○	○	○	○	○
Trends in Narcan administration at scenes of overdoses	○	○	○	○	○
Overdose statistics by age and gender	○	○	○	○	○
Overdose statistics by geographic area	○	○	○	○	○
Locations of free Narcan in Cayuga County	○	○	○	○	○
Locations of treatment and recovery services in Cayuga County	○	○	○	○	○
Locations of medication drop boxes in Cayuga County	○	○	○	○	○

Part 3

We would now like to better understand your user experience with the Cayuga County Dashboard Overdose Dashboard. If you wish to review the dashboard prior to answering the below questions, you can find the link to the dashboard here: https://www.cayugacounty.us/1634/Overdose-Data-Reports-Trends.

7. Based on your experience using Cayuga County Overdose Data Dashboard, how much do you agree or disagree with each of the following statements?

	Strongly agree	Agree	Neither agree nor disagree	Disagree	Strongly disagree
FEATURES AND NAVIGATION					
1. The visuals have enough descriptive text for me to understand what they are showing.	○	○	○	○	○
2. The interactive nature of the dashboard makes it easy for me to find the information that I am looking for.	○	○	○	○	○
3. The types of data portrayed on the dashboard are useful.	○	○	○	○	○
4. The dashboard is easy to find on the Cayuga County website.	○	○	○	○	○
5. The dashboard webpage takes too long to load.	○	○	○	○	○
INFORMATION ON RESOURCES					
1. Based on the information provided on the dashboard, I can easily find and access Narcan if needed.	○	○	○	○	○
2. Based on the information provided on the dashboard, I can easily find and access treatment and recovery services if needed.	○	○	○	○	○
3. Based on the information provided on the dashboard, I can easily find and access medication drop boxes if needed.	○	○	○	○	○

8. Based on your experience using Cayuga County Overdose Data Dashboard, how much do you agree or disagree with each of the following statements?

	Strongly agree	Agree	Somewhat agree	Neither agree nor disagree	Somewhat disagree	Disagree	Strongly disagree
**USER EXPERIENCE**							
1. The data dashboard is easy to use.	○	○	○	○	○	○	○
2. The functionalities of the data dashboard fulfill my needs.	○	○	○	○	○	○	○

9. Do you intend to visit the Cayuga County Overdose Data Dashboard in the future?

Yes
No

10. How likely are you to recommend Cayuga County Overdose Data Dashboard to a friend or colleague?

Not at all likely					Neutral					Extremely likely
0	1	2	3	4	5	6	7	8	9	10

11. What prevents you from accessing and/or using the Cayuga County Overdose Data Dashboard more frequently?

____

12. How can we improve the dashboard?

____

Part 4

In the last part of this survey, please tell us about yourself. This helps us understand who is accessing and using the Cayuga County Overdose Data Dashboard.

13. What is your age (in years)?

____

14. Which gender do you identify as?

    Male
    Female
    Non-binary
    Other, please specify ____

15. Which race/ethnicity do you identify as?

    Black or African American
    Hispanic or Latino
    American Indian or Alaska Native
    Asian
    Native Hawaiian or Other Pacific Islander
    Middle Eastern or North African
    Other, please specify ____

16. What is the highest degree or level of school you have completed?

    Some high school, but no degree
    High school diploma or GED
    Associate degree
    Bachelor's degree
    Post-graduate degree

17. Do you live or work in Cayuga County?

    Yes
    No

18. Where do you live?

    City or town ____
    State ____
    Zip code ____

19. Please select the option(s) that best describes you (check all that apply):

    A person with lived experience of opioid/substance use
    A family member or friend of someone with lived experience of opioid/substance use
    A healthcare provider
    A first responder
    An individual who works with organization(s) that address mental health and/or substance use
    A community member
    Other (please specify)
